# Specific dynamic of serum procalcitonin in critically ill patients affected by Gram-negative bacilli septic thrombophlebitis

**DOI:** 10.1186/s13054-018-2116-8

**Published:** 2018-07-23

**Authors:** Martina Spaziante, Giancarlo Ceccarelli, Samir Al Moghazi, Francesco Alessandri, Mario Venditti

**Affiliations:** 1 Department of Public Health and Infectious Diseases, University of Rome Sapienza, Viale del Policlinico 155, Rome, Italy; 2 Azienda Policlinico Umberto I, Rome, Italy; 3 Department of Anesthesia and Intensive Care Medicine, University of Rome Sapienza, Rome, Italy

We read with interest the study by Thomas-Rüddel et al. [1] evaluating the influence of specific pathogens and different foci of infections on serum procalcitonin (PCT) concentrations. The authors concluded that PCT levels were higher in patients with Gram-negative bacteremia compared with patients with Gram-positive or fungal diseases, whereas urogenital and abdominal foci of infection were associated with twofold increased PCT values, independent of causative pathogen. Unfortunately, this study did not provide data on PCT trends in patients affected by endovascular infections.

We recently collected a small series of 13 cases of endovascular infections caused by thrombophlebitis due to Gram-negative bacilli (GNB) in the intensive care unit (ICU) of a large University Hospital in Italy. The mean age of patients enrolled was 59.2 ± 13.6 years with a predominance of male sex (61.5%); the mean SAPS II at the admission was 39.7 ± 8.1 points and the most frequent cause of ICU admission was a recent polytrauma (84.6%). All patients had persistent bacteremia despite administration of in vitro active antibiotics and removal of intravascular devices. The diagnosis of septic thrombophlebitis was corroborated by CT scan (53.8%) or echodoppler (46.2%), and thrombus appositions mainly involved aortic trunks (61.5%). The blood isolates were four *Klebsiella pneumoniae*, four *Acinetobacter baumannii*, one *Enterobacter spp.*, one *Pseudomonas aeruginosa*, one *Morganella morganii*, one *Providencia rettgeri*, and one *Klebsiella oxytoca*.

Despite the prolonged duration of bacteremia and the appropriate antibiotic therapy, all patients showed an indolent clinical course, with no multi-organ failure, prompt clinical improvement, and rapid decrease of plasma PCT concentrations within normal ranges after the onset of septic episodes (Fig. 1).

**Fig. 1:**
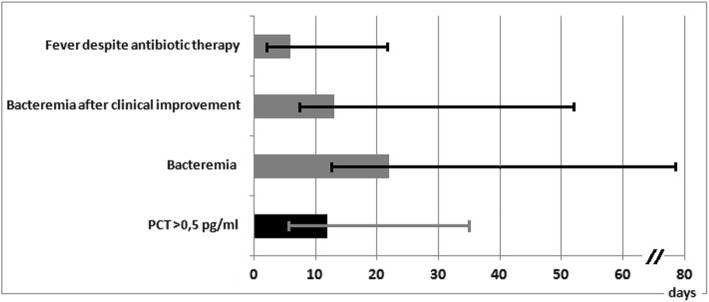
Clinical and microbiological course of GNB septic thrombophlebitis in critically ill patients. Clinical improvement was defined as withdrawal of inotropic support and fever < 38 °C. The median values (with range) are shown

As previously reported, PCT is produced in response to inflammatory cytokines and bacterial endotoxins [2]. In our cases the rapid decrease of PCT, followed by a stable normalization of serum concentration despite persistence of bacteremia, could be explained with the well-known mechanism of immune tolerance: in fact the selective blocks of some pro-inflammatory pathways, activated by bacterial endotoxins or cytokines, could impact on the production of PCT and favor a long indolent clinical course, even in the face of microbial eradication failure [3].

In conclusion, we think that our data could contribute to complete the results of Thomas-Rüddel et al. and are worthy of being further investigated in a larger series of cases. Physicians should be aware that serum PCT measurements should be interpreted with caution in assessing the clinical course of GNB endovascular infections. In this setting performance of serial blood cultures seems to remain the most adequate guide to therapy [1, 4].

## Abbreviations

CT scan: Computed tomography scan
GNB: Gram-negative bacilli
ICU: Intensive care unit
PCT: Procalcitonin
SAPS II: Simplified Acute Physiology Score
